# General practitioners’ barriers to cross-sectoral collaboration on pregnant women with vulnerabilities: a cross-sectional survey in Danish general practice

**DOI:** 10.1080/02813432.2024.2432371

**Published:** 2024-12-08

**Authors:** Louise Brygger Venø, Line Bjørnskov Pedersen, Jens Søndergaard, Ruth Kirk Ertmann, Dorte Ejg Jarbøl

**Affiliations:** a Department of Public Health, Research Unit of General Practice, University of Southern Denmark, Odense, Denmark; b Department of Public Health, DaCHE – Danish Centre for Health Economics, University of Denmark, Odense, Denmark; c Department of Public Health, Research Unit of General Practice, University of Copenhagen, Copenhagen, Denmark

**Keywords:** General practitioner, barriers, antenatal care, collaboration, vulnerability

## Abstract

**Background:**

Pregnancy vulnerability contributes to poor perinatal mental health. Proper cross-sectoral collaboration may mitigate perinatal mental health problems. General practitioners (GPs) often face barriers when assessing pregnancy vulnerability, but little is known about GPs’ perceived barriers to the cross-sectoral collaboration on vulnerable pregnant women.

**Objective:**

To explore GPs’ barriers to cross-sectoral collaboration on pregnant women with vulnerabilities, and how barriers are associated with the organization of antenatal care (ANC) and general practice characteristics.

**Design, setting and subjects:**

A cross-sectional questionnaire study among Danish GPs (*n* = 3465).

**Main outcome measures:**

Descriptive statistics according to the Theoretical Domains Framework describes the barriers to collaboration. Analytical statistics with ordered logistic regression models demonstrate associations between selected barriers (the main outcome measures) and organization of ANC, GP and practice, characteristics.

**Results:**

A total of 760 GPs (22%) participated. Perceived GP barriers to collaboration were lacking knowledge of ANC levels relevant to vulnerable pregnant women, insufficient information on vulnerability indicators from collaborating parties, heavy workload and insufficient remuneration for collaborative meetings. Being young were associated with insufficient GP knowledge of ANC levels. Old age was associated with less likelihood of experiencing heavy workload as a barrier.

**Conclusions:**

Barriers to collaboration on vulnerable pregnant women were associated with some GP-organizational characteristics including low experience in collaborating with partners in ANC, and GP characteristics, such as age. Reducing general practice workload, e.g. by reorganizing priority areas, releasing more time to vulnerable patients, and improving cross-sectoral information sharing on vulnerability might improve collaboration on vulnerable pregnant women.

## Introduction

According to the World Health Organization (WHO), between 10 and 20% of all pregnant women are vulnerable [1]. Pregnancy vulnerability has been defined in terms of psychosocial problems, i.e. preexisting psychiatric diseases [2–4], and social determinants, i.e. women from migrant background who are poorly integrated [4], young age, low socioeconomic status, adverse childhood experiences or exposure to partner violence [5,6]. The consequence of undetected and unsupported pregnancy vulnerability may be an increased risk of antenatal or postnatal depression or anxiety [2,7], fetal growth restriction, preterm birth [3,7,8] and attachment disorders in the child [8,9].

In Denmark, general practitioners (GPs) are the first line contact to the antenatal care (ANC) system, and they are responsible [4] for evaluating the risks and resources of the pregnant women and their families [10]. Special preventive efforts are dedicated to vulnerable pregnant women, involving cross-sectoral collaboration between partners in the health care system, i.e. GP, hospital obstetricians, midwives, other medical specialists and the municipal social care system [10]. The role of the Danish ANC and its collaborating partners has been previously described [11,12]. A Danish report points to a deficit in the detection of pregnancy vulnerability since less than 25% of women with severe pregnancy vulnerability were identified in family practice [13]. In the similar UK health care system only 50% of women with perinatal depression are identified in general practice [14].

Barriers to cross-sectoral collaboration have been studied regarding vulnerable patients in general (i.e. patients of all ages), pointing to insufficient cross-sectoral communication, not understanding the roles of others, and not prioritizing the needed time to participate in cross-sectoral meetings [15–17]. Inconsistent teamwork between GPs and partners in the ANC system is a critical barrier hindering proper assessment and cross-sectoral collaboration regarding vulnerable pregnant women [14,18–20], and experts in health innovation call for attention to clinicians’ perceived complexity of collaborating across systems [21].

The Theoretical Domains Framework (TDF) applies to identify influences of behaviors, and barriers to implementation problems in both qualitative and quantitative designs [22]. We have qualitatively explored the GP’s perceived complexity of assessing and collaborating on pregnancy vulnerability within the frame of the TDF [11,12]. Conditional barriers such as time constraints and heavy workload, and structural barriers with the lack of shared information on social vulnerability across sectors [12], and improper cross-sectoral communication pathways [11] limited the GP’s assessment and collaboration on pregnancy vulnerability. This questionnaire study contributes by quantifying the GP’s perceived barriers to the cross-sectoral collaboration on vulnerable pregnant women illustrated by the TDF. The aim of this study is to (1) describe the extent of GPs’ perceived barriers in the cross-sectoral collaboration on vulnerable pregnant women and (2) analyze the associations between the predefined GP-perceived barriers and (a) organizational characteristics of ANC in general practice and (b) GP and practice characteristics.

## Materials and methods

In Denmark, 98% of citizens are registered with a GP, and use of the healthcare system is tax-funded and free of charge for the patient [23]. Among Danish GPs, 94% are self-employed and responsible for managing ANC consultations and engaging in the cross-sectoral collaboration, whereas 6% are salaried without this responsibility (i.e. locum GPs and GPs employed in regional or non-union clinics). The self-employed GPs contract with the public funder and the contracts include a fee schedule with reimbursable services, including a special fee for ANC consultations (25–68 Euro) and for participating in cross-sectoral collaborative meetings (864 Euro/hour).

We conducted a cross-sectional survey among Danish self-employed GPs (*N* = 3465). Locum GPs and GPs employed in regional, or non-union clinics were excluded. The study was designed to assess the barriers to collaboration according to the 14 psychological behavioral domains of the TDF [24] (Table 1).

**Table 1.** Theoretical Domains Framework (TDF).

TDF domain^a^	TDF label in the article	TDF domains included in the article^b^
Knowledge	A	Included
Skills	B	Included
Memory and attention	C	Included
Environmental context and resources	D	Included
Beliefs about capabilities	E	Included
Reinforcement	F	Included
Goals	G	Included
Social influences		Excluded
Social/professional role and identity		Excluded
Beliefs about consequences		Excluded
Behavioral regulation		Excluded
Intention		Excluded
Optimism		Excluded
Emotion		Excluded

^a^ TDF: Theoretical Domains Framework, 14 domains version according to Cane et al. [24].

^b^ See codebook in Appendix 1 for elaboration.

We developed and tested the questionnaire in five consecutive steps resumed below, to achieve content and face validity, acceptability and feasibility.

1. Literature search on the definition of the constructs: pregnancy vulnerability and barriers to cross-sectoral collaboration on vulnerable pregnant women.
2. Five focus group interviews with 20 GPs, analyzed with deductive thematic coding to the TDF. Results are published elsewhere [11,12,25].
3. Operationalization of the constructs and items based on discussions in the clinically experienced research group, and central findings from the literature search and qualitative TDF-based findings [11]. Inspiration was taken from a validated TDF questionnaire [24]. Constructs relating to seven of the 14 TDF domains were selected, with belonging items exploring barriers to collaboration with different cross-sectoral partners (Appendix 1).
4. Seven cognitive pilot tests to test time consumption, content validity, acceptability and feasibility.
5. A field test among 12 GPs, indicating the use of all response categories.

The final questionnaire measured the GP’s barriers to collaboration on women with pregnancy vulnerability, grouped by seven TDF domains, distributed on nine constructs and 17 items. Responses were scored on five-point Likert scales (from fully agree to fully disagree), and a category of ‘don’t know/not relevant’ was included. The questionnaire further contained items on ANC organization in general practice and practice characteristics. The questionnaire is shown in Appendix 2. Information on GP characteristics (age and gender), and further practice characteristics (practice type and location) were collected from publicly available registers (Appendix 1).

We invited the GPs to participate in the online survey between October 2021 and February 2022 by postal letters and posted two reminder letters to non-respondents.

Descriptive statistics were applied to describe the ANC organization, the GP and practice characteristics and to compare the characteristics of non-responders on available data. The barriers to cross-sectoral collaboration regarding vulnerable pregnant women were described according to the TDF domains and constructs.

From the descriptive findings, we selected four constructs as outcome variables for regression analyses, measuring barriers to collaboration; the four outcome variables are ‘limited knowledge of ANC levels’, ‘low self-efficacy in distinguishing the proper ANC level’, ‘workload limiting resources for collaboration’ and ‘remuneration not motivating collaboration’. The selection of barriers were based on criteria of clinical relevance, i.e. aspects of the GPs’ role in ANC (knowledge and self-efficacy), workload and motivation, variation in responses and potential for behavior change, e.g. through continuous medical education. We investigated the associations between the four barriers and the following explanatory variables: ANC organization in general practice ((I) allocated time to the first ANC consultation, (II) delegating ANC consultations to staff members, (III) prioritization of provider-patient continuity in ANC consultations, (IV) allocating extra time to vulnerable women and (V) collaboration with other health care providers, i.e. health care visitors, social obstetricians, municipal family department), GP-characteristics (gender and age) and general practice characteristics ((I) practice type, (II) number of fulltime capacities, (III) patient load, i.e. number of patients per full-time GP capacity and (IV) region), using four separate univariable and multivariable ordered logistic regression models. In the multivariable ordered logistic regression models, we adjusted analyses for all explanatory variables to account for possible confounding. Standard errors were modeled as cluster robust at the general practice level to account for correlation between GPs from the same general practice. ‘Don’t know/not relevant’ responses on the barrier measures were omitted from the analysis. Missing data on GP characteristics (four missing values on age) and practice characteristics (nine missing values) were imputed by allocation to the most frequent response categories.

## Results

**Figure 1.**
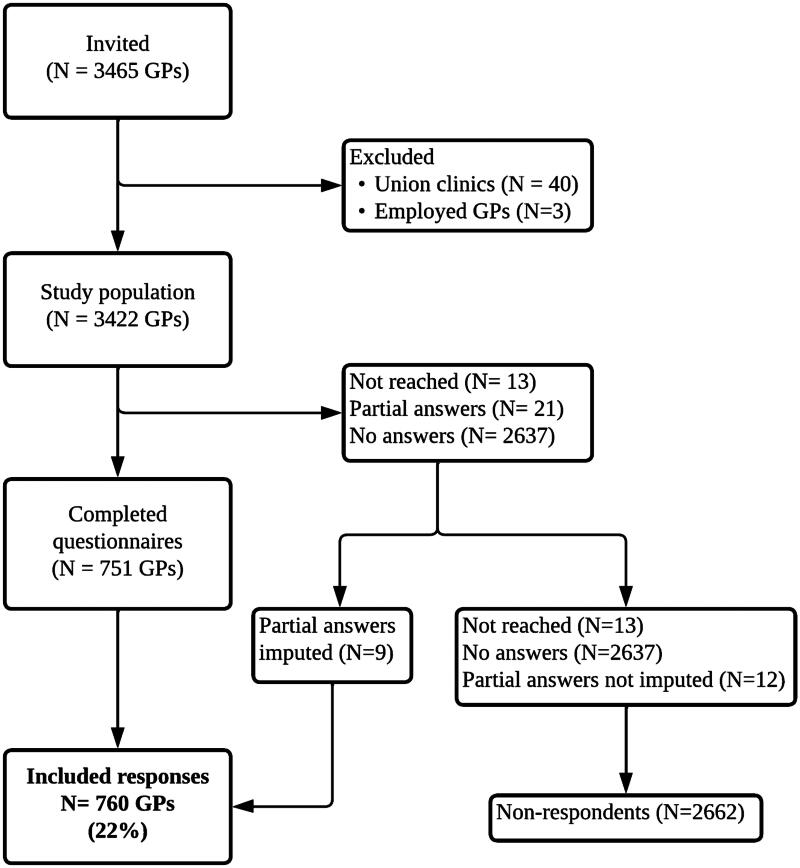
Flow of participants.

Among 3465 invited GPs, responses from 760 GPs (22%) were included in this study. The flowchart is shown in Figure 1.

The descriptive statistics of ANC organization, GP and practice characteristics are shown in Table 2. Compared to non-responders, the responding GPs were roughly evenly distributed across gender and age. Slightly fewer GPs above 60 years of age from single-handed practices and the Capital Region responded to the survey. Most GPs were organized in partnership practices (84.3%), with 2–4 full-time capacities (65.1%), and a moderate patient load (73.4%). Most GPs allocated 30 min or more to the first ANC consultation (91%). Almost half of the GPs never or rarely (49.2%) allocated extra time to ANC for vulnerable pregnant women. One in five of GPs (20%) delegated ANC consultations entirely to practice staff. Most GPs reported that they often or always (92.1%) prioritize continuity in ANC. Many GPs reported never or rarely collaborating with healthcare visitors (57.5%), social obstetric outpatient units (27.6%) and municipal family departments (61.7%).

**Table 2.** Descriptive statistics of general practice characteristics, and antenatal care organization.

Explanatory variables	Category	Respondents (%)	Non-respondents (%)
	Total	760 (100.0)	2662 (100%)
ANC organization			
Time to 1st ANC consultation in minutes	<30 min	68 (8.9)	N/A
	30 min	347 (45.7)	N/A
	>30 min	345 (45.4)	N/A
Delegating ANC consultations fully to practice staff members	No	608 (80.0)	N/A
	Yes	152 (20.0)	N/A
Prioritizing continuity in ANC between HCP and pregnant woman	Never	8 (1.1)	N/A
	Rarely	13 (1.7)	N/A
	Sometimes	39 (5.1)	N/A
	Often	494 (65.0)	N/A
	Always	206 (27.1)	N/A
Allocating extra time to ANC for vulnerable pregnant women	Never	144 (18.9)	N/A
	Rarely	230 (30.3)	N/A
	Sometimes	189 (24.9)	N/A
	Often	132 (17.4)	N/A
	Always	65 (8.6)	N/A
Collaborating with healthcare visitors	Never	140 (18.4)	N/A
	Rarely	297 (39.1)	N/A
	Sometimes	179 (23.6)	N/A
	Often	106 (13.9)	N/A
	Always	38 (5.0)	N/A
Collaborating with social obstetric outpatient units	Never	42 (5.5)	N/A
	Rarely	168 (22.1)	N/A
	Sometimes	182 (23.9)	N/A
	Often	195 (25.7)	N/A
	Always	173 (22.8)	N/A
Collaborating with municipal family departments	Never	106 (13.9)	N/A
	Rarely	363 (47.8)	N/A
	Sometimes	216 (28.4)	N/A
	Often	52 (6.8)	N/A
	Always	23 (3.0)	N/A
GP and practice characteristics			
GP gender	Males	330 (43.4)	10,830 (40.7)
	Females	430 (56.6)	1579 (59.3)
GP age in years	≤45	210 (27.6)	578 (21.7)
	>45 and ≤60	422 (55.5)	1424 (53.5)
	>60	128 (16.8)	660 (24.8)
Practice type	Single-handed	119 (15.7)	545 (20.5)
	Partnership	641 (84.3)	2117 (79.5)
Number of full-time capacities	1	176 (23.2)	N/A
	2–4	495 (65.1)	N/A
	>5	89 (11.7)	N/A
Patient load (number of patients/GP)	<1500	122 (16.1)	N/A
	1500–2000	558 (73.4)	N/A
	>2000	80 (10.5)	N/A
Region	Capital Region	193 (25.4)	879 (33.0)
	Region Zealand	87 (11.4)	351 (13.2)
	Region of Southern Denmark	208 (27.4)	587 (22.1)
	Central Denmark Region	215 (28.3)	596 (22.4)
	Region of Northern Denmark	57 (7.5)	249 (9.3)

GP: general practitioners; ANC: antenatal care; HCP: healthcare professionals; N/A: not answered.

For respondents, there were four missing values on GP age and nine missing values on practice characteristics. For non-respondents, there were two missing values on gender, 18 on GP age and two on practice type. Missing data for responders and non-responders were allocated to the most frequent response categories.

Descriptive results and barriers to collaboration on vulnerable pregnant women according to the TDF domains and constructs, are shown in Table 3. In the following, the TDF domains are illustrated with capital letters, and the domain-specific items with numbers.

**Table 3.** Descriptive barriers/non-barriers to the cross-sectoral collaboration on vulnerable pregnant women.

	TDF domain	Construct	Item	Fully agree or agree	Neither agree nor disagree	Disagree or fully disagree	Don’t know/not relevant	*N* (%)
A	Knowledge	Limited knowledge of antenatal care levels	Lack of procedural knowledge – of ANC levels (including cross-sectoral collaboration)	295 (38.8%)	155 (20.4%)	303 (39.9%)	7 (0.9%)	760 (100%)
B	Skills	Competence	Lack of competence in distinguishing ANC levels	199 (26.2%)	210 (27.6%)	345 (45.4%)	6 (0.8%)	760 (100%)
C.1.1	Memory attention and decision-making *(reversed)*	GP attention	Perceiving to be attentive to collaborative opportunities with health visitors	565 (74.3%)	98 (12.9%)	90 (11.8%)	7 (0.9%)	760 (100%)
C.1.2	Memory attention and decision-making	GP attention	Perceiving to be attentive to collaborative opportunities with social obstetric	674 (88.7%)	52 (6.8%)	26 (3.4%)	8 (1.1%)	760 (100%)
C.1.3	Memory attention and decision-making	GP attention	Perceiving to be attentive to collaborative opportunities with municipal social workers	515 (67.8%)	142 (18.7%)	89 (11.7%)	14 (1.8%)	760 (100%)
C.2.1^a^	Memory attention and decision-making *(reversed)*	Staff members attention	The staff is attentive to collaborative opportunities with health visitors	102 (67.1%)	21 (13.8%)	21 (13.8%)	8 (5.3%)	152 (100%)^a^
C.2.2^a^	Memory attention and decision-making	Staff members attention	The staff is attentive to collaborative opportunities with social obstetric units	121 (79.6%)	20 (13.2%)	7 (4.6%)	4 (2.6%)	152 (100%)^a^
C.2.3^a^	Memory attention and decision-making	Staff members attention	The staff is attentive to collaborative opportunities with municipal social workers	93 (61.2%)	32 (21.1%)	18 (11.8%)	9 (5.9%)	152 (100%)^a^
D.1	Environmental context and resources	Workload limiting resources for collaboration	The workload in general practice is limiting resources for collaboration	410 (53.9%)	166 (21.8%)	178 (23.4%)	6 (0.8%)	760 (100%)
D.2.1	Environmental context and resources	Limited available information from cross-sectoral collaborators	Vulnerability assessment is limited due to a lack of information from hospital outpatient clinics	241 (31.7%)	211 (27.8%)	286 (37.6%)	22 (2.9%)	760 (100%)
D.2.2	Environmental context and resources	Limited available information from cross-sectoral collaborators	Vulnerability assessment is limited due to the lack of information from private practicing specialists	262 (34.5%)	216 (28.4%)	246 (32.4%)	36 (4.7%)	760 (100%)
D.2.3	Environmental context and resources	Limited available information from cross-sectoral collaborators	Vulnerability assessment is limited due to the lack of information from psychologists	395 (52.0%)	195 (25.7%)	121 (15.9%)	49 (6.4%)	760 (100%)
D.2.4	Environmental context and resources	Limited available information from cross-sectoral collaborators	Vulnerability assessment is limited due to the lack of information from health visitors	337 (44.3%)	202 (26.6%)	206 (27.1%)	15 (2.0%)	760 (100%)
D.2.5	Environmental context and resources	Limited available information from cross-sectoral collaborators	Vulnerability assessment is limited due to the lack of information from municipal social workers	467 (61.4%)	181 (23.8%)	88 (11.6%)	24 (3.2%)	760 (100%)
E.1	Believes about capabilities	Low self-efficacy	Perceiving it difficult to distinguish the proper ANC level (including cross-sectoral collaboration) to vulnerable women	273 (35.9%)	202 (26.6%)	280 (36.8%)	5 (0.7%)	760 (100%)
F.1	Reinforcement *(reversed)*	Remuneration motivating collaboration	Remuneration motivating collaboration	112 (14.7%)	294 (38.7%)	307 (40.4%)	47 (6.2%)	760 (100%)
G.1	Goal	Goal	Other collaborative task have higher priority than ANC collaboration	64 (8.4%)	222 (29.2%)	466 (61.3%)	8 (1.1%)	760 (100%)

TDF: Theoretical Domains Framework; ANC: antenatal care.

^a^ *N* = 152 as only GPs who delegated ANC fully to staff members received this question. To comply with general data protection requirements for cells with small numbers (<5), categories for fully agree/agree and fully disagree/disagree are combined, while the category don’t know/not relevant is omitted from the table.

A total of 38.8% fully agreed or agreed with the item ‘lack of knowledge of the content of ANC levels’ (A), and 26.2% fully agreed or agreed with ‘having difficulties distinguishing them’ (B). In general, the GPs perceived themselves as being attentive to collaborating with health visitors, social obstetric outpatient units and municipal family departments (C.1.1–C.1.3). GPs delegating ANC to staff members generally perceived staff as attentive to cross-sectoral collaboration (C.2.1–C.2.3).

Well over half of the GPs (53.9%) perceived the workload in general practice to limit resources for collaboration (D.1). Many GPs perceived vulnerability assessment as limited due to a lack of information from different cross-sectoral collaborators (D.2.1–D.2.5), i.e. hospital outpatient clinics (31.7%), private practising specialists (34.5%), psychologists (52.0%), health care visitors (44.3%) and social workers from municipal family departments (61.4%). Low self-efficacy in distinguishing the proper care to vulnerable women was perceived among 35.9% of the GPs (E.1). Related to reinforcement, 40.4% fully disagreed or disagreed, that the collective fee, i.e. the remuneration, motivated collaboration (F.1) and finally, did 8.4% fully agree or agree that other collaborative tasks than ANC collaboration had higher priority (G.1).

Analyses of association between outcome measures – i.e. barriers to collaboration, and characteristics of the ANC organization in the clinics, GP and practice characteristics, included the four barriers: lacking knowledge of ANC levels (A), perceived high workload in the clinics (D.1) self-inefficacy (E.1) and lacking reinforcement (F.1). The results from the adjusted multivariable analyses models are shown in Table 4. The following results present adjusted OR with 95% CI. Unadjusted analyses appear in Appendix 3.

**Table 4.** Adjusted associations between barriers to cross-sectoral collaboration and GP characteristics, practice characteristics and ANC characteristics (*N* = 760).

Outcome variable	TDF-domain	Knowledge (A)	Believes about capabilities (E.1)	Environmental context and resources (D.1)	Reinforcement (F.1)
	Construct	Limited knowledge of ANC levels	Low self-efficacy	Workload limiting resources for collaboration	Remuneration motivating collaboration (*reversed*)
	Item	Lack of procedural knowledge of ANC levels including cross-sectoral collaboration)	Perceiving it difficult to distinguish the proper ANC level (including cross-sectoral collaboration) to vulnerable women	The workload in general practice is limiting resources for collaboration	Remuneration motivating collaboration (*reversed*)
*Explanatory variable*		adjOR^a^ (CI)	adjOR^a^ (CI)	adjOR^a^ (CI)	adjOR^a^ (CI)
Organizational characteristics of ANC					
Time to 1st ANC consultation in minutes^a^	<30	0.95 (0.58–1.56)	0.79 (0.45–1.37)	1.02 (0.64–1.65)	1.17 (0.73–1.90)
	30	1.00 (1.00–1.00)	1.00 (1.00–1.00)	1.00 (1.00–1.00)	1.00 (1.00–1.00)
	>30	0.80 (0.61–1.06)	0.73 (0.55–0.97)*	1.02 (0.77–1.35)	0.83 (0.63–1.11)
Delegating ANC consultations fully to practice staff^a^	No	1.00 (1.00–1.00)	1.00 (1.00–1.00)	1.00 (1.00–1.00)	1.00 (1.00–1.00)
	Yes	0.75 (0.54–1.05)	0.94 (0.66–1.34)	1.30 (0.88–1.92)	1.18 (0.85–1.65)
Prioritizing continuity in ANC between HCP and pregnant woman^a^	Never	0.54 (0.09–3.27)	0.83 (0.11–6.05)	1.86 (0.08–42.25)	2.00 (0.39–10.35)
	Rarely	0.88 (0.34–2.31)	1.13 (0.43–2.94)	1.08 (0.49–2.40)	0.50 (0.17–1.52)
	Sometimes	1.00 (1.00–1.00)	1.00 (1.00–1.00)	1.00 (1.00–1.00)	1.00 (1.00–1.00)
	Often	1.25 (0.71–2.20)	1.35 (0.67–2.71)	1.40 (0.78–2.51)	1.40 (0.78–2.50)
	Always	0.92 (0.50–1.72)	1.18 (0.56–2.46)	1.01 (0.54–1.89)	1.46 (0.78–2.73)
Allocating extra time to vulnerable pregnant women^a^	Never	1.16 (0.74–1.80)	1.22 (0.79–1.89)	0.98 (0.62–1.55)	0.96 (0.62–1.49)
	Rarely	1.28 (0.90–1.82)	1.32 (0.93–1.86)	1.04 (0.74–1.46)	1.10 (0.75–1.62)
	Sometimes	1.00 (1.00–1.00)	1.00 (1.00–1.00)	1.00 (1.00–1.00)	1.00 (1.00–1.00)
	Often	1.14 (0.75–1.71)	1.05 (0.69–1.60)	1.09 (0.73–1.63)	1.02 (0.68–1.53)
	Always	0.80 (0.45–1.41)	0.84 (0.44–1.62)	0.80 (0.46–1.37)	1.03 (0.58–1.84)
Collaborating with health care visitors^a^	Never	2.20 (1.42–3.41)***	2.26 (1.44–3.56)***	1.80 (1.15–2.83)*	0.84 (0.53–1.33)
	Rarely	1.51 (1.06–2.14)*	1.70 (1.19–2.43)**	1.51 (1.05–2.17)*	0.97 (0.68–1.37)
	Sometimes	1.00 (1.00–1.00)	1.00 (1.00–1.00)	1.00 (1.00–1.00)	1.00 (1.00–1.00)
	Often	0.95 (0.62–1.47)	1.06 (0.69–1.64)	1.05 (0.67–1.64)	0.62 (0.40–0.95)*
	Always	0.99 (0.53–1.86)	1.15 (0.58–2.26)	1.05 (0.53–2.07)	0.77 (0.35–1.72)
Collaborating with social obstetricians^a^	Never	3.48 (1.58–7.65)**	2.57 (1.03–6.41)*	1.72 (0.84–3.56)	2.05 (0.88–4.80)
	Rarely	1.03 (0.68–1.56)	1.19 (0.78–1.80)	1.52 (0.99–2.34)	1.30 (0.85–2.01)
	Sometimes	1.00 (1.00–1.00)	1.00 (1.00–1.00)	1.00 (1.00–1.00)	1.00 (1.00–1.00)
	Often	1.17 (0.80–1.70)	1.19 (0.83–1.72)	1.26 (0.87–1.82)	0.96 (0.65–1.41)
	Always	0.95 (0.63–1.41)	0.83 (0.55–1.24)	0.80 (0.53–1.22)	1.13 (0.76–1.66)
Collaborating with municipal family department^a^	Never	0.55 (0.34–0.91)*	0.69 (0.43–1.11)	0.96 (0.58–1.58)	1.17 (0.72–1.91)
	Rarely	1.11 (0.81–1.52)	1.06 (0.77–1.45)	1.20 (0.87–1.67)	1.19 (0.85–1.67)
	Sometimes	1.00 (1.00–1.00)	1.00 (1.00–1.00)	1.00 (1.00–1.00)	1.00 (1.00–1.00)
	Often	1.51 (0.88–2.58)	1.81 (1.08–3.02)*	1.39 (0.72–2.68)	0.84 (0.49–1.41)
	Always	0.94 (0.35–2.50)	0.75 (0.29–1.95)	0.93 (0.45–1.92)	0.70 (0.27–1.82)
GP characteristics					
GP gender^a^	Male	1.30 (0.99–1.71)	1.20 (0.90–1.60)	1.23 (0.92–1.63)	1.10 (0.85–1.43)
	Female	1.00 (1.00–1.00)	1.00 (1.00–1.00)	1.00 (1.00–1.00)	1.00 (1.00–1.00)
GP age in years^a^	≤45	1.39 (1.01–1.93)*	1.43 (1.00–2.06)	1.21 (0.87–1.69)	0.78 (0.57–1.07)
	46–60	1.00 (1.00–1.00)	1.00 (1.00–1.00)	1.00 (1.00–1.00)	1.00 (1.00–1.00)
	>60	0.96 (0.67–1.36)	1.20 (0.86–1.68)	0.45 (0.31–0.66)***	0.70 (0.47–1.03)
General practice characteristics					
Practice type^a^	Single-handed	1.19 (0.69–2.05)	1.08 (0.63–1.84)	0.93 (0.56–1.54)	0.73 (0.45–1.20)
	Partnership	1.00 (1.00–1.00)	1.00 (1.00–1.00)	1.00 (1.00–1.00)	1.00 (1.00–1.00)
Number of fulltime capacities^a^	1	1.18 (0.73–1.90)	1.15 (0.74–1.79)	0.72 (0.47–1.11)	1.20 (0.76–1.90)
	2–4	1.00 (1.00–1.00)	1.00 (1.00–1.00)	1.00 (1.00–1.00)	1.00 (1.00–1.00)
	5 or more	0.89 (0.54–1.44)	0.96 (0.63–1.47)	0.86 (0.58–1.29)	1.01 (0.65–1.59)
Patient load (average number of patients/GP)^a^	<1500	0.90 (0.62–1.32)	0.96 (0.64–1.44)	0.72 (0.49–1.05)	0.73 (0.51–1.05)
	1500–2000	1.00 (1.00–1.00)	1.00 (1.00–1.00)	1.00 (1.00–1.00)	1.00 (1.00–1.00)
	>2000	0.78 (0.45–1.35)	0.71 (0.43–1.16)	0.50 (0.30–0.84)**	0.84 (0.50–1.41)
Region^a^	Capital Region	0.80 (0.54–1.18)	0.82 (0.56–1.20)	1.09 (0.73–1.62)	1.17 (0.80–1.70)
	Region Zealand	0.60 (0.37–0.96)*	0.50 (0.33–0.77)**	0.76 (0.47–1.24)	0.80 (0.47–1.38)
	Region of Southern Denmark	1.00 (1.00–1.00)	1.00 (1.00–1.00)	1.00 (1.00–1.00)	1.00 (1.00–1.00)
	Central Denmark Region	0.72 (0.51–1.03)	0.74 (0.51–1.09)	0.96 (0.67–1.39)	0.87 (0.62–1.21)
	Region of Northern Denmark	0.81 (0.42–1.55)	0.91 (0.52–1.57)	0.82 (0.42–1.62)	0.98 (0.55–1.76)

GP: general practitioner; ANC: antenatal care; CI: 95% confidence interval; OR: odds ratio; adj: adjusted.

* *p* < .05.

** *p* < .01.

*** *p* < .001.

^a^ Adjusted for all explanatory variables (organizational characteristics in practice, GP and practice characteristics).

### Organization of ANC and associations with barriers to collaboration

Allocating more than 30 min to the first ANC consultation (compared to 30 min) was associated with a lower likelihood of perceiving it difficult to distinguish the proper ANC level (OR 0.73 (0.55–0.97)).

Never/rarely collaborating with healthcare visitors was associated with a higher likelihood of having limited knowledge of ANC levels (OR 2.20 (1.42–3.41)/OR 1.51 (1.06–2.14)) and perceived difficulties in distinguishing ANC levels (OR 2.26 (1.44–3.56)/OR 1.70 (1.19–2.43)). Also, never/rarely collaborating with healthcare visitors was associated with a higher likelihood of perceiving a heavy workload to limit resources for collaboration (OR 1.80 (1.15–2.83)/OR 1.51 (1.05–2.17)). Similar associations were found between never collaborating with social-obstetricians and lacking knowledge of ANC levels (OR 3.48 (1.58–7.65)) and perceived difficulties in distinguishing ANC levels (OR 2.57 (1.03–6.41)).

Often collaborating with healthcare visitors was associated with a lower likelihood of perceiving remuneration as a motivator for collaboration (OR 0.62 (0.40–0.95)).

Never collaborating with municipal family departments was associated with a lower likelihood of lacking knowledge of ANC levels (OR 0.55 (0.34–0.91)), whereas often collaborating with municipal family departments was associated with a higher likelihood of perceived self-inefficiency in distinguishing ANC levels (OR 1.81 (1.08–3.02)).

### GP and practice characteristics and associations with barriers to collaboration

Younger GP age, compared to 46–60 years of age, was associated with a higher likelihood of lacking knowledge of ANC levels (OR 1.39 (1.01–1.93)). Moreover, being +60 years was associated with a lower likelihood of perceiving a heavy workload to limit collaboration (OR 0.45 (0.31–0.66)). GPs with a higher patient load, compared to a moderate patient load, had a lower likelihood of perceiving a heavy workload as a barrier to collaboration (OR 0.50 (0.30–0.84)).

## Discussion

### Principal findings

This cross-sectional survey described several GP-reported barriers to the cross-sectoral collaboration on vulnerable pregnant women, i.e. insufficient knowledge of ANC levels and inefficacy in distinguishing the proper ANC level, heavy workload limiting resources for collaboration, insufficient cross-sectoral information sharing on vulnerability indicators, and the remuneration system not motivating collaboration. We found associations between organizational characteristics such as not collaborating with partners in antenatal and municipal care, and barriers of insufficient knowledge and self-inefficacy in distinguishing ANC levels, and workload limiting resources for collaboration. GP characteristics such as young GP age were associated with the barrier of insufficient knowledge of ANC levels, whereas high GP age was associated with less likelihood of perceived workload limitations.

### Strength and weaknesses

The TDF as a model is aimed at understanding barriers in health professional behavior, and therefore the application of TDF as a theoretical model is a strength. The questionnaire items were designed from qualitative TDF-guided findings on barriers to cross-sectoral collaboration regarding vulnerable pregnant women [11], which ensured in-depth elaboration of the barriers. The main limitation of this study is the small sample size, which may be attributable to the increasing workload due to Covid-19 vaccination in general practice during the sampling period. The small sample size increases the risk of sampling bias. Also, the responding GPs may have had a more than average interest in vulnerable patients, and therefore the perceived barriers may be downward biased, consequently affecting the generalizability of the findings. The self-reported data might have included some declaration biases, such as recall bias, i.e. GP perceive consultations with vulnerable pregnant women as rare [4], but it may also be influenced by other parameters, or cultural variation, e.g. minor structural differences in the cross-sectoral collaboration on vulnerable pregnant women between the regions. Also, the self-reported responses are prone to desirability bias. However, we sought to minimize this with a questionnaire introductory text justifying the ambiguity of the vulnerability concept and thoroughly explaining our aim. The findings represent the GPs perspectives, and studies exploring the perspectives of other ANC collaborators are needed to fully illustrate barriers in the cross-sectoral collaboration. The limited sample size resulted in wide confidence intervals and, consequently, greater uncertainties around the findings. Despite these limitations, we have no reason to believe that the established associations between GP barriers, and ANC organization, GP, and practice characteristics are invalid.

### Findings in relation to other studies

Experts in health innovation have called for knowledge about clinicians perceived complexity of collaborating across systems [21]. British, Irish and Danish qualitative studies [11,12,14,18], and register studies [26] found that GPs feel unsupported when dealing with women with pregnancy vulnerability or perinatal mental health issues, due to poor joint communication in cross-sectoral collaboration. This survey’s findings contribute by quantifying the extent of GPs perceived poor joint communication on vulnerability indicators with the different collaborators in ANC, and by exploring possible associations with ANC organization, GP and practice characteristics. Our findings indicate a problem of lacking GP attention to pregnancy vulnerability, and that many GPs therefore fail to allocate extra time to ANC for possibly vulnerable women. However, the GPs may have a certain knowledge about their patients’ degree of vulnerability due to the high degree of continuity. Nevertheless, allocating extra time to ANC to possibly vulnerable women seems important, since this study found an association with a higher GP self-efficacy in distinguishing the right level of antenatal support. The barriers of limited GP knowledge and self-inefficacy in distinguishing ANC levels, and GP perceived limited cross-sectoral information sharing on pregnancy vulnerability are in line with prior qualitative findings [12,27]. The association between the barrier of perceived high workload and low experience with cross-sectoral collaboration is supported by a survey study on GPs’ job stress in 11 high-income countries comparable to the Danish setting, in which an association was found between heavy workload, job-stress and time constraints, and the lack of communication and coordination among healthcare sectors [28]. Also, a Norwegian qualitative study points to GPs’ perceptions that the increasing need for cross-sectoral collaboration on patients with complex issues contributes to increased workload in general practice [29].

A Norwegian study explored GPs’ participation in multidisciplinary meetings and found that patients with psychological disorders generally motivated meeting attendance, and that young age of the GP, and a short patient list predicted higher meeting attendance [30]. On the contrary, we found that a high patient load was associated with a lower likelihood of perceiving heavy workload as a barrier to cross-sectoral ANC collaboration. This may indicate that the ability to tackle a large patient list and the extent of collaboration may have a common explanation, e.g. GP’s attitude and the organization of the daily work schedule.

Seemingly, the cross-sectoral collaboration on vulnerable pregnant women is to a lesser extent influenced by monetary incentives, since GPs who reported often collaborating with health visitors, had less likelihood of perceiving remunerations as a motivation for collaboration. This is supported by a Danish cross-sectional study, showing heterogeneity in GPs’ work motivation [31].

### Meaning of the study

The findings indicate that GPs are limited in their work of assessing pregnancy vulnerability, due to collaborative barriers such as insufficient knowledge of ANC levels, heavy workload and the lack of shared information on pregnancy vulnerabilities across sectors.

Young GPs might benefit from continuous medicinal education activities on the consequences of pregnancy vulnerability, knowledge about the ANC levels, and the most optimal ANC collaboration regarding vulnerable women.

Policymakers might consider enhancing proper cross-sectoral information sharing on vulnerability indicators regarding pregnant women and families to support GPs in assessing pregnancy vulnerability. Also, the findings may support policymakers in reorganizing priority areas to accommodate the workload in general practice and release more time for the GPs to care for vulnerable patients.

Interventional studies targeting the identified GP barriers to collaboration are needed to explore whether increased cross-sectoral collaboration and shared information support GPs in assessing vulnerable pregnant women or vulnerable patients in general.

## Supplementary Material

Appendix 2_English questionnaire_collaboration regarding vulnerable pregnant women.docx

Appendix 3_Table 5_unadjusted analyses_revised.docx

Appendix 1_Codebook_barriers to cross_sectoral collaboration.docx

## Data Availability

The datasets generated and analyzed in the current study are not publicly available due to the data protection regulations of the Danish Data Protection Agency. Access to data is strictly limited to the researchers who have obtained permission for data processing. This permission was given to the Research Unit of General Practice, Department of Public Health, University of Southern Denmark.
