# Supplementary material for: General practitioners’ barriers to cross-sectoral collaboration on pregnant women with vulnerabilities: a cross-sectional survey in Danish general practice
Source: Scand J Prim Health Care. 2024 Dec 8;43(2):292–302. doi: 10.1080/02813432.2024.2432371 (PMC12090315; doi:10.1080/02813432.2024.2432371)
Supplement: Appendix 2_English questionnaire_collaboration regarding vulnerable pregnant women.docx [file IPRI_A_2432371_SM8138.docx]

English questionnaire

# Project goal

This study aims to examine the work of GPs with vulnerable pregnant women.

The questionnaire was developed after qualitative interviews with general practitioners about barriers to assessing, collaborating and reporting on vulnerable pregnant women. The questions are inspired by theories of behavior and motivation - including experience, working conditions and belief in one's ability to act.

In your answer, please consider as a starting point how you manage the care of vulnerable pregnant patients as you meet them throughout the pregnancy.

Vulnerability can be seen in varying degrees in pregnant women with, e.g. psychiatric illness, psychological or social problems and substance abuse problems. However, vulnerability may also be less clear. We have a particular focus on vulnerability that can affect the mother’s or the couple's ability to provide care for the future child.

### **Part 1: Antenatal care in your practice**

Initially, we ask about the organization of pregnancy consultations in your / your practice.

Are there pregnant women in your practice who are only seen by the clinic staff and not by the doctor at the first pregnancy examination?

(1) ❑ Yes

(0) ❑ No

Indicate which type of clinic staff independently handles pregnancy examinations in your practice (excluding GP trainees)? Tick one or more boxes

(1) ❑ Nurses

(2) ❑ Midwives

(3) ❑ Health care assistants

(4) ❑ Secretaries

(5) ❑ Employed medical students

(6) ❑ Other

Do you have GP trainees in your clinic (phase 1-4)?

(1) ❑ Yes

(0) ❑ No

How often is it the GP trainee that conducts the 1^st^ pregnancy examinations in your clinic?

(1) ❑ Always

(2) ❑ Often

(3) ❑ Some time

(4) ❑ Rarely

(5) ❑ Never

How often does the same doctor or clinic staff see the pregnant woman at most pregnancy examinations? (1st, 2nd and 3rd pregnancy examinations, as well as the postpartum examination)

(1) ❑ Always

(2) ❑ Often

(3) ❑ Some time

(4) ❑ Rarely

(5) ❑ Never

How much time do you usually set aside for the 1st pregnancy examination (excluding urine sample and blood sampling)? If parts of the pregnancy examination have been delegated, state the total time.

(1) ❑ 15 min or below

(2) ❑ 20 min

(3) ❑ 30 min

(4) ❑ 40 min

(5) ❑ 45 min

(6) ❑ 50 min

(7) ❑ 60 min

How often is extra time set aside for the first pregnancy examination if you know in advance that a pregnant woman is vulnerable?

(1) ❑ Always

(2) ❑ Often

(3) ❑ Some time

(4) ❑ Rarely

(5) ❑ Never

The following questions are about your general routines for cross-sectoral collaboration in antenatal care and reporting on vulnerable pregnant women.

Cross-sectoral collaboration means collaboration with health nurses, Social workers in the municipalities' family department and the obstetric outpatient clinics.

How often do you contact the following partners when you see a vulnerable pregnant woman in your practice? This question does not concern reporting.

|  | Always | Often | Some time | Rarely | Never |
| --- | --- | --- | --- | --- | --- |
| Health care visitors | (1) ❑ | (2) ❑ | (3) ❑ | (4) ❑ | (5) ❑ |
| Social obstetric outpatient clinics | (1) ❑ | (2) ❑ | (3) ❑ | (4) ❑ | (5) ❑ |
| Social workers in the municipalities’ family department | (1) ❑ | (2) ❑ | (3) ❑ | (4) ❑ | (5) ❑ |

How often do you choose to report to the municipality when you become concerned that a pregnant woman is vulnerable to a degree which may affect her ability to provide care for the future child?

If you do not remember a situation where reporting a vulnerable pregnant woman was relevant, select "not relevant".

(1) ❑ Always

(2) ❑ Often

(3) ❑ Some time

(4) ❑ Rarely

(5) ❑ Never

(99) ❑ Not relevant

**(Part 2: Assessment of vulnerable pregnant women – not shown. It is related to another article)**

**Part 3: The cross-sectoral collaboration on vulnerable pregnant women**

The following questions are about your experience of cross-sectoral collaboration on vulnerable pregnant women.

We point out that some of the previous questions are repeated, but now with a focus on collaboration.

How much do you agree or disagree with the following?

|  | Fully agree | Agree | Neither agree nor disagree | Disagree | Fully disagree | Don’t know/ not relevant |
| --- | --- | --- | --- | --- | --- | --- |
| I lack knowledge about the content of the four levels of antenatal care (i.e., basic offer, expanded basic offer, cross-sectoral collaboration, collaboration with the social obstetric department) | (5) ❑ | (4) ❑ | (3) ❑ | (2) ❑ | (1) ❑ | (99) ❑ |
| I often doubt which level of antenatal care I should refer the pregnant women. | (5) ❑ | (4) ❑ | (3) ❑ | (2) ❑ | (1) ❑ | (99) ❑ |

How much do you agree or disagree with the following?

I’m aware of the possibility of collaborating with the following partners on vulnerable pregnant women.

|  | Fully agree | Agree | Neither agree nor disagree | Disagree | Fully disagree | Don’t know/ not relevant |
| --- | --- | --- | --- | --- | --- | --- |
| Health care visitors | (1) ❑ | (2) ❑ | (3) ❑ | (4) ❑ | (5) ❑ | (99) ❑ |
| Social obstetric outpatient clinics | (1) ❑ | (2) ❑ | (3) ❑ | (4) ❑ | (5) ❑ | (99) ❑ |
| Social workers in the municipal family department | (1) ❑ | (2) ❑ | (3) ❑ | (4) ❑ | (5) ❑ | (99) ❑ |

My/our clinic staff are aware of the possibility of collaborating with the following partners on vulnerable pregnant women:

|  | Fully agree | Agree | Neither agree nor disagree | Disagree | Fully disagree | Don’t know/ not relevant |
| --- | --- | --- | --- | --- | --- | --- |
| Health care visitors | (1) ❑ | (2) ❑ | (3) ❑ | (4) ❑ | (5) ❑ | (99) ❑ |
| Social obstetric outpatient clinics | (1) ❑ | (2) ❑ | (3) ❑ | (4) ❑ | (5) ❑ | (99) ❑ |
| Social workers in the municipal family department | (1) ❑ | (2) ❑ | (3) ❑ | (4) ❑ | (5) ❑ | (99) ❑ |

Assessment of vulnerable pregnant women is limited by lack of information (outpatient epicrises /correspondence) from the following cross-sectoral collaborators:

|  | Fully agree | Agree | Neither agree nor disagree | Disagree | Fully disagree | Don’t know/ not relevant |
| --- | --- | --- | --- | --- | --- | --- |
| Hospital outpatient clinics (e.g. psychiatric outpatient clinics) | (5) ❑ | (4) ❑ | (3) ❑ | (2) ❑ | (1) ❑ | (99) ❑ |
| Private specialists (e.g. private psychiatrists) | (5) ❑ | (4) ❑ | (3) ❑ | (2) ❑ | (1) ❑ | (99) ❑ |
| Psychologists | (5) ❑ | (4) ❑ | (3) ❑ | (2) ❑ | (1) ❑ | (99) ❑ |
| Health care visitors | (5) ❑ | (4) ❑ | (3) ❑ | (2) ❑ | (1) ❑ | (99) ❑ |
| Social workers in the municipal family department | (5) ❑ | (4) ❑ | (3) ❑ | (2) ❑ | (1) ❑ | (99) ❑ |

How much do you agree or disagree with the following?

|  | Fully agree | Agree | Neither agree nor disagree | Disagree | Fully disagree | Don’t know/ not relevant |
| --- | --- | --- | --- | --- | --- | --- |
| The total number of tasks in general practice limits my/our time for collaboration with cross-sectoral partners in antenatal care. | (5) ❑ | (4) ❑ | (3) ❑ | (2) ❑ | (1) ❑ | (99) ❑ |
| I find it difficult to distinguish to which of the four levels of antenatal care the pregnant women belong (i.e., basic offer, expanded basic offer, cross-sectoral collaboration, collaboration with the social obstetric department) | (5) ❑ | (4) ❑ | (3) ❑ | (2) ❑ | (1) ❑ | (99) ❑ |
| Other collaborative tasks (i.e. on patients with chronic illnesses) have a higher priority for me than collaborating cross-sectoral on vulnerable pregnant women. | (5) ❑ | (4) ❑ | (3) ❑ | (2) ❑ | (1) ❑ | (99) ❑ |
| The collective fee provides motivation to participate in cross-sectoral meetings (DKK 138.87 per 10 min) | (5) ❑ | (4) ❑ | (3) ❑ | (2) ❑ | (1) ❑ | (99) ❑ |

**(Part 4: Reporting on vulnerable pregnant women – not shown. It is related to another article)**

**Part 5: Your Practice**

Finally, we have some general questions about you and your practice. We also ask for your thoughts on possible new initiatives on the topic.

Is your practice a solo or partnership practice?

(1) ❑ Solo practice

(2) ❑ Partnership practice

Do you work in collaborative practice?

(1) ❑ Yes

(0) ❑ No

Do you work in a shared practice?

(1) ❑ Yes

(0) ❑ No

How many doctors work in your practice? (incl. GP owners, locum GPs. GP trainees do not count)

(1) ❑ 1 doctor (myself)

(2) ❑ 2 doctors

(3) ❑ 3 doctors

(4) ❑ 4 doctors

(5) ❑ 5 doctors

(6) ❑ 6 doctors

(7) ❑ 7 doctors or more

How many full-time capacities are associated with your practice number?

(1) ❑ 1 full-time capacity

(2) ❑ 2 full-time capacities

(3) ❑ 3 full-time capacities

(4) ❑ 4 full-time capacities

(5) ❑ 5 full-time capacities

(6) ❑ 6 full-time capacities

(7) ❑ 7 full-time capacities or more

Enter how many patients are registered within your practice (enter the number for the entire practice)

_______

How many clinic staff have you employed in your practice (incl. Staff in any collaborative practice)? Enter the number of staff employed (GP trainees do not count)

If there was a continuing medical education offer on the topics below, would you choose to participate?

|  | Yes | No | Don’t know |
| --- | --- | --- | --- |
| Assessment of vulnerable pregnant women | (1) ❑ | (0) ❑ | (2) ❑ |
| Cross-sectoral collaboration in antenatal care for vulnerable pregnant women | (1) ❑ | (0) ❑ | (2) ❑ |

Do you want activities that strengthen your local collaboration with the municipalities' family department on vulnerable pregnant women?

(1) ❑ Yes

(0) ❑ No

(2) ❑ Don’t know

Do you wish for a clinical guideline on the following subjects?

|  | Yes | No | Don’t know |
| --- | --- | --- | --- |
| Assessment of vulnerable pregnant women | (1) ❑ | (0) ❑ | (2) ❑ |
| Cross-sectoral collaboration in antenatal care for vulnerable pregnant women | (1) ❑ | (0) ❑ | (2) ❑ |

Thank you for your participation in the questionnaire survey
