# Supplementary material for: General practitioners’ barriers to cross-sectoral collaboration on pregnant women with vulnerabilities: a cross-sectional survey in Danish general practice
Source: Scand J Prim Health Care. 2024 Dec 8;43(2):292–302. doi: 10.1080/02813432.2024.2432371 (PMC12090315; doi:10.1080/02813432.2024.2432371)
Supplement: Appendix 3_Table 5_unadjusted analyses_revised.docx [file IPRI_A_2432371_SM8137.docx]

Table 5. Association between barriers to cross-sectoral collaboration and GP characteristics, practice characteristics, and ANC characteristics. Unadjusted analyses

| **Outcome variable** | **TDF-domain** | Knowledge (A) | Believes about capabilities (E.1) | Environmental context and resources (D.1) | Reinforcement (F.1) |
| --- | --- | --- | --- | --- | --- |
|  | **Construct** | Limited knowledge of ANC levels | Low self-efficacy | Workload limiting resources for collaboration | Remuneration motivating collaboration (*reversed*) |
| **Explanatory variable** | **Text** | Unadj OR (CI) | Unadj OR (CI) | Unadj OR (CI) | Unadj OR (CI) |
| Time to 1st ANC consultation in minutes | <30 | 1.13 (0.71-1.81) | 0.86 (0.52-1.42) | 1.00 (0.64-1.57) | 1.15 (0.74-1.79) |
|  | 30 | 1.00 (1.00-1.00) | 1.00 (1.00-1.00) | 1.00 (1.00-1.00) | 1.00 (1.00-1.00) |
|  | >30 | 0.82 (0.63-1.07) | 0.74 (0.57-0.97)* | 1.10 (0.84-1.45) | 0.86 (0.65-1.12) |
| Delegating ANC consultations fully to practice staff | No | 1.00 (1.00-1.00) | 1.00 (1.00-1.00) | 1.00 (1.00-1.00) | 1.00 (1.00-1.00) |
|  | Yes | 0.68 (0.50-0.94)* | 0.84 (0.60-1.15) | 1.22 (0.86-1.71) | 1.08 (0.77-1.51) |
| Prioritizing continuity in ANC between HCP and pregnant woman | Never | 0.66 (0.13-3.41) | 1.19 (0.22-6.44) | 1.35 (0.13-14.42) | 1.64 (0.31-8.64) |
|  | Rarely | 0.72 (0.28-1.88) | 1.16 (0.41-3.24) | 1.29 (0.58-2.88) | 0.61 (0.18-2.01) |
|  | Sometimes | 1.00 (1.00-1.00) | 1.00 (1.00-1.00) | 1.00 (1.00-1.00) | 1.00 (1.00-1.00) |
|  | Often | 1.00 (0.58-1.70) | 1.13 (0.60-2.15) | 1.08 (0.61-1.92) | 1.21 (0.68-2.13) |
|  | Always | 0.80 (0.45-1.43) | 1.04 (0.53-2.03) | 0.64 (0.35-1.18) | 1.25 (0.69-2.26) |
| Allocating extra time to vulnerable pregnant women | Never | 1.25 (0.82-1.93) | 1.23 (0.81-1.89) | 1.19 (0.78-1.81) | 1.09 (0.72-1.63) |
|  | Rarely | 1.40 (1.01-1.94)* | 1.36 (0.98-1.88) | 1.18 (0.85-1.64) | 1.24 (0.86-1.78) |
|  | Sometimes | 1.00 (1.00-1.00) | 1.00 (1.00-1.00) | 1.00 (1.00-1.00) | 1.00 (1.00-1.00) |
|  | Often | 1.07 (0.73-1.57) | 0.93 (0.63-1.38) | 0.98 (0.68-1.40) | 1.06 (0.72-1.56) |
|  | Always | 0.85 (0.48-1.51) | 0.85 (0.46-1.56) | 0.67 (0.39-1.16) | 1.08 (0.62-1.86) |
| Collaborating with health care visitors | Never | 2.14 (1.43-3.18)*** | 2.32 (1.55-3.49)*** | 1.89 (1.26-2.83)** | 1.04 (0.71-1.53) |
|  | Rarely | 1.56 (1.13-2.17)** | 1.78 (1.28-2.49)*** | 1.61 (1.16-2.23)** | 1.04 (0.74-1.46) |
|  | Sometimes | 1.00 (1.00-1.00) | 1.00 (1.00-1.00) | 1.00 (1.00-1.00) | 1.00 (1.00-1.00) |
|  | Often | 0.97 (0.64-1.49) | 1.10 (0.71-1.70) | 1.11 (0.72-1.70) | 0.60 (0.40-0.90)* |
|  | Always | 0.84 (0.45-1.56) | 0.94 (0.48-1.85) | 0.89 (0.49-1.64) | 0.70 (0.34-1.47) |
| Collaborating with social obstetricians | Never | 2.90 (1.46-5.76)** | 2.48 (1.08-5.66)* | 1.42 (0.74-2.73) | 1.82 (0.93-3.59) |
|  | Rarely | 1.15 (0.79-1.70) | 1.37 (0.94-2.00) | 1.44 (0.97-2.14) | 1.31 (0.87-1.97) |
|  | Sometimes | 1.00 (1.00-1.00) | 1.00 (1.00-1.00) | 1.00 (1.00-1.00) | 1.00 (1.00-1.00) |
|  | Often | 1.10 (0.78-1.55) | 1.22 (0.86-1.72) | 1.28 (0.92-1.79) | 0.88 (0.61-1.27) |
|  | Always | 0.88 (0.61-1.28) | 0.84 (0.57-1.22) | 0.74 (0.50-1.08) | 0.99 (0.70-1.40) |
| Collaborating with municipal family department | Never | 1.06 (0.68-1.65) | 1.21 (0.79-1.84) | 1.39 (0.89-2.17) | 1.40 (0.94-2.09) |
|  | Rarely | 1.31 (0.98-1.75) | 1.27 (0.94-1.70) | 1.44 (1.07-1.95)* | 1.23 (0.90-1.68) |
|  | Sometimes | 1.00 (1.00-1.00) | 1.00 (1.00-1.00) | 1.00 (1.00-1.00) | 1.00 (1.00-1.00) |
|  | Often | 1.30 (0.81-2.10) | 1.54 (0.97-2.45) | 1.10 (0.59-2.04) | 0.70 (0.45-1.11) |
|  | Always | 0.67 (0.27-1.65) | 0.49 (0.22-1.13) | 0.67 (0.35-1.28) | 0.73 (0.30-1.74) |
| GP gender | Male | 1.28 (0.99-1.66) | 1.22 (0.94-1.59) | 0.99 (0.76-1.29) | 1.05 (0.82-1.35) |
|  | Female | 1.00 (1.00-1.00) | 1.00 (1.00-1.00) | 1.00 (1.00-1.00) | 1.00 (1.00-1.00) |
| GP age in years | ≤ 45 | 1.42 (1.05-1.93)* | 1.51 (1.07-2.12)* | 1.33 (0.97-1.82) | 0.81 (0.60-1.09) |
|  | 46-60 | 1.00 (1.00-1.00) | 1.00 (1.00-1.00) | 1.00 (1.00-1.00) | 1.00 (1.00-1.00) |
|  | >60 | 1.08 (0.77-1.52) | 1.32 (0.95-1.84) | 0.43 (0.31-0.60)*** | 0.70 (0.50-0.99)* |
| Practice type | Single-handed | 1.14 (0.80-1.63) | 1.08 (0.75-1.55) | 0.54 (0.38-0.77)*** | 0.92 (0.65-1.29) |
|  | Partnership | 1.00 (1.00-1.00) | 1.00 (1.00-1.00) | 1.00 (1.00-1.00) | 1.00 (1.00-1.00) |
| Number of fulltime capacities | 1 | 1.09 (0.80-1.50) | 1.10 (0.81-1.49) | 0.54 (0.40-0.73)*** | 1.13 (0.83-1.55) |
|  | 2-4 | 1.00 (1.00-1.00) | 1.00 (1.00-1.00) | 1.00 (1.00-1.00) | 1.00 (1.00-1.00) |
|  | 5 or more | 1.00 (0.65-1.54) | 1.10 (0.75-1.61) | 0.87 (0.59-1.26) | 1.01 (0.67-1.52) |
| Patient load (average number of patients/GP) | <1500 | 1.00 (0.71-1.40) | 1.04 (0.72-1.51) | 0.68 (0.48-0.96)* | 0.75 (0.54-1.04) |
|  | 1500-2000 | 1.00 (1.00-1.00) | 1.00 (1.00-1.00) | 1.00 (1.00-1.00) | 1.00 (1.00-1.00) |
|  | >2000 | 0.92 (0.56-1.53) | 0.80 (0.51-1.27) | 0.49 (0.32-0.76)** | 0.94 (0.59-1.50) |
| Region | Capital Region | 0.83 (0.58-1.20) | 0.83 (0.58-1.20) | 1.06 (0.74-1.54) | 1.21 (0.85-1.73) |
|  | Region Zealand | 0.65 (0.41-1.03) | 0.54 (0.35-0.81)** | 0.84 (0.53-1.33) | 0.87 (0.53-1.42) |
|  | Region of Southern Denmark | 1.00 (1.00-1.00) | 1.00 (1.00-1.00) | 1.00 (1.00-1.00) | 1.00 (1.00-1.00) |
|  | Central Denmark Region | 0.68 (0.49-0.96)* | 0.73 (0.51-1.04) | 1.05 (0.74-1.49) | 0.88 (0.64-1.21) |
|  | Region of Northern Denmark | 0.77 (0.40-1.46) | 0.87 (0.51-1.48) | 0.74 (0.42-1.30) | 0.97 (0.55-1.70) |
| GP=general practitioner, ANC= Antenatal care, CI = 95% confidence interval *** p<0,001, ** p <0,01, * p<0,05 | | | | | |
