# Supplementary material for: General practitioners’ barriers to cross-sectoral collaboration on pregnant women with vulnerabilities: a cross-sectional survey in Danish general practice
Source: Scand J Prim Health Care. 2024 Dec 8;43(2):292–302. doi: 10.1080/02813432.2024.2432371 (PMC12090315; doi:10.1080/02813432.2024.2432371)
Supplement: Appendix 1_Codebook_barriers to cross_sectoral collaboration.docx [file IPRI_A_2432371_SM8136.docx]

# Codebook: Collaboration regarding vulnerable pregnant women

| **Variable name** | **Category** | **Data type** | **Definition** | **Operationalization (values)** |
| --- | --- | --- | --- | --- |
| **Explanatory variables** | | | | |
| **Practice characteristics (* indicate that variables are not shown in tables)** | | | | |
| Region | Practice region | Register | The Danish Health Data Network (MedCom) | Capital (1), Zealand (2), Southern Denmark (3), Central Denmark (4), Northern Denmark (5) |
| Pre-defined practice type | Predefined practice type | Register | The Danish eHealth Portal (Sundhed.dk) | Solo (1), Partnership (2) |
| Number of GPs * | Number of GPs in practice | Self-reported |  | Numbers 1 (1), 2 (2), 3 (3), 4 (4), 5 (5), 6 (6), 7 or more (7) |
| Number of full-time capacities | Number of full-time capacities | Self-reported |  | Numbers 1 (1), 2 (2), 3 (3), 4 (4), 5 (5), 6 (6), 7 or more (7) |
| Number of patients in practice* | Number of patients in the practice | Self-reported |  | Numbers |
| Number of practice staff * | Number of practice staff | Self-reported |  | Numbers |
| Patient load | Patient load | Self-reported | Number of patients/numbers of full-time capacities | < 1500 (1), 1500-2000 (2), > 2000 (3) |
| **GP characteristics** | | | | |
| Gender | Gender | Register | The Danish Professional Registry | Male (0), Female (1) |
| Age | Age | Register | The Danish Professional Registry | date |
| **ANC organizational characteristics** | | | | |
| Delegation to practice staff | Delegating ANC full to practice staff | Self-reported |  | Yes (1), No (0) |
| Type of practice staff conducting ANC consultations* | Type of practice staff conducting ANC consultations | Self-reported |  | Nurses (1), Midwives (2), Health care assistants (3), Secretaries (4), Employed medical students (5), others (6) |
| Other staff * | If other staff conducts ANC consultation, specify which other staff | Self-reported |  | Letters |
| GP trainees * | GP trainees in practice | Self-reported |  | Yes (1), No (0) |
| GP trainees doing 1^st^ ANC consultation | Frequency of GP trainees conducting ANC consultations | Self-reported | How often is it the GP trainee that conducts the 1^st^ pregnancy examinations in your clinic? | Always (1), Often (2), Sometimes (3), Rarely (4), Never (5) |
| Prioritizing continuity in ANC | Frequency of prioritizing continuity in ANC consultations between HCP and pregnant women | Self-reported | How often does the same GP or practice staff consult the pregnant woman at most ANC consultations? | Always (1), Often (2), Sometimes (3), Rarely (4), Never (5) |
| Time to 1st ANC consultation in minutes | Time to 1st ANC consultation in minutes | Self-reported | The total amount of time allocated to 1st ANC consultation | ≤ 15 min (1), 20 min (2), 30 min (3), 40 min (4), 45 min (5), 50 min (6), 60 min (7) |
| Allocating extra time to ANC for vulnerable pregnant women | Frequency of allocating extra time to 1st ANC consultation | Self-reported | Allocating extra time to pregnant women with a known vulnerability | Always (1), Often (2), Sometimes (3), Rarely (4), Never (5) |
| Collaborating with health visitors | Frequency of collaborating with health care nurses | Self-reported | When consulting a vulnerable pregnant woman – the frequency of contacting healthcare nurses | Always (1), Often (2), Sometimes (3), Rarely (4), Never (5) |
| Collaborating with social obstetric outpatient clinics | Frequency of collaborating with social-obstetric outpatient clinics | Self-reported | When consulting a vulnerable pregnant woman – the frequency of contacting social obstetric outpatient clinics | Always (1), Often (2), Sometimes (3), Rarely (4), Never (5) |
| Collaborating with municipal social services | Frequency of collaborating with municipal social services | Self-reported | When consulting a vulnerable pregnant woman – the frequency of contacting the municipal social services | Always (1), Often (2), Sometimes (3), Rarely (4), Never (5) |
| **Outcome variables** | | | | |
| **GP reported barriers to collaboration regarding vulnerable pregnant women according to TDF domains (* indicate that variables are not shown in tables)** | | | | |
| **TDF domain** | **Construct** | **Data type** | **Item** | **Operationalization (values)** |
| Knowledge | Knowledge- factual | Self-reported | Lack of procedural knowledge - of antenatal care levels | Fully agree (5), Agree (4), Neither agree nor disagree (3), Disagree (2), Fully disagree (1), don't know/not relevant (99) |
| Skills | Skills - competence | Self-reported | Lack of competence in distinguishing antenatal care levels | Fully agree (5), Agree (4), Neither agree nor disagree (3), Disagree (2), Fully disagree (1), don't know/not relevant (99) |
| Memory and attention (*reversed*) | GP attention – health visitors | Self-reported | Perceiving to be attentive to collaborative opportunities with health visitors | Fully agree (1), Agree (2), Neither agree nor disagree (3), Disagree (4), Fully disagree (5), don't know/not relevant (99) |
| Memory and attention (*reversed*) | GP attention – social obstetricians | Self-reported | Perceiving to be attentive to collaborative opportunities with social obstetricians | Fully agree (1), Agree (2), Neither agree nor disagree (3), Disagree (4), Fully disagree (5), don't know/not relevant (99) |
| Memory and attention (*reversed*) | GP attention – municipal social workers | Self-reported | Perceiving to be attentive to collaborative opportunities with municipal social workers | Fully agree (1), Agree (2), Neither agree nor disagree (3), Disagree (4), Fully disagree (5), don't know/not relevant (99) |
| Memory and attention (*reversed*) | staff members attention – health visitors | Self-reported | Perceiving to be attentive to collaborative opportunities with health visitors | Fully agree (1), Agree (2), Neither agree nor disagree (3), Disagree (4), Fully disagree (5), don't know/not relevant (99) |
| Memory and attention (*reversed*) | staff members attention – social obstetricians | Self-reported | Perceiving to be attentive to collaborative opportunities with social obstetric | Fully agree (1), Agree (2), Neither agree nor disagree (3), Disagree (4), Fully disagree (5), don't know/not relevant (99) |
| Memory and attention (*reversed*) | staff members attention – municipal social workers | Self-reported | Perceiving to be attentive to collaborative opportunities with municipal social workers | Fully agree (1), Agree (2), Neither agree nor disagree (3), Disagree (4), Fully disagree (5), don't know/not relevant (99) |
| Behavioral regulation | Behavioral regulation | Excluded | Overlapping with the domains memory and attention | none |
| Environmental context and resources | workload | Self-reported | The workload in general practice is limiting resources for collaboration | Fully agree (5), Agree (4), Neither agree nor disagree (3), Disagree (2), Fully disagree (1), don't know/not relevant (99) |
| Environmental context and resources | limited available information from hospital outpatient clinics | Self-reported | Vulnerability assessment is limited due to lacking information from hospital outpatient clinics | Fully agree (5), Agree (4), Neither agree nor disagree (3), Disagree (2), Fully disagree (1), don't know/not relevant (99) |
| Environmental context and resources | limited available information from private practicing specialist | Self-reported | Vulnerability assessment is limited due to lacking information from private practicing specialists | Fully agree (5), Agree (4), Neither agree nor disagree (3), Disagree (2), Fully disagree (1), don't know/not relevant (99) |
| Environmental context and resources | limited available information from psychologists | Self-reported | Vulnerability assessment is limited due to lacking information from psychologists | Fully agree (5), Agree (4), Neither agree nor disagree (3), Disagree (2), Fully disagree (1), don't know/not relevant (99) |
| Environmental context and resources | limited available information from health visitors | Self-reported | Vulnerability assessment is limited due to lacking information from health visitors | Fully agree (5), Agree (4), Neither agree nor disagree (3), Disagree (2), Fully disagree (1), don't know/not relevant (99) |
| Environmental context and resources | limited available information from municipal social workers | Self-reported | Vulnerability assessment is limited due to lacking information from municipal social workers | Fully agree (5), Agree (4), Neither agree nor disagree (3), Disagree (2), Fully disagree (1), don't know/not relevant (99) |
| Social support | record keeping | Excluded | Perceived giving invalid answers.  e.g. being too difficult to answer | none |
| Social and professional role and identity | Professional role | Excluded | Perceived giving invalid answers.  e.g. being too difficult to answer | none |
| Believe in capability | Self-efficacy | Self-reported | Perceiving it difficult to distinguish the proper antenatal care level to vulnerable women. | Fully agree (5), Agree (4), Neither agree nor disagree (3), Disagree (2), Fully disagree (1), don't know/not relevant (99) |
| Believe in consequences | Believe in consequences | Excluded | Perceived giving invalid answers.  e.g. being too difficult to answer | none |
| Intention | Intention | Excluded | Perceived giving invalid answers.  e.g. being too difficult to answer | none |
| Reinforcement *(reversed)* | Reinforcement - incentive | Self-reported | Remuneration motivating collaboration | Fully agree (1), Agree (2), Neither agree nor disagree (3), Disagree (4), Fully disagree (5), don't know/not relevant (99) |
| Goal | Priority | Self-reported | Other collaborative task have higher priority than ANC collaboration | Fully agree (5), Agree (4), Neither agree nor disagree (3), Disagree (2), Fully disagree (1), don't know/not relevant (99) |
| Emotion | Emotion | Excluded | Perceived giving invalid answers | none |
| Optimism | Optimism | Excluded | Perceived giving invalid answers | none |
